# The Spinal Transcriptome after Cortical Stroke: In Search of Molecular Factors Regulating Spontaneous Recovery in the Spinal Cord

**DOI:** 10.1523/JNEUROSCI.2571-18.2019

**Published:** 2019-06-12

**Authors:** Julia Kaiser, Martina Maibach, Iris Salpeter, Niels Hagenbuch, Vladimir B.C. de Souza, Mark D. Robinson, Martin E. Schwab

**Affiliations:** ^1^Brain Research Institute and Department of Health Sciences and Technology, University and ETH Zurich, 8057, Zurich, Switzerland,; ^2^Institute for Epidemiology, Biostatistics, and Prevention, Department of Biostatistics, University of Zurich, 8001, Zurich, Switzerland, and; ^3^Department of Molecular Life Sciences and SIB Swiss Institute of Bioinformatics, University of Zurich, 8052, Zurich, Switzerland

**Keywords:** plasticity, recovery, regeneration, spinal cord, stroke, transcriptome

## Abstract

In response to cortical stroke and unilateral corticospinal tract degeneration, compensatory sprouting of spared corticospinal fibers is associated with recovery of skilled movement in rodents. To date, little is known about the molecular mechanisms orchestrating this spontaneous rewiring. In this study, we provide insights into the molecular changes in the spinal cord tissue after large ischemic cortical injury in adult female mice, with a focus on factors that might influence the reinnervation process by contralesional corticospinal neurons. We mapped the area of cervical gray matter reinnervation by sprouting contralesional corticospinal axons after unilateral photothrombotic stroke of the motor cortex in mice using anterograde tracing. The mRNA profile of this reinnervation area was analyzed using whole-genome sequencing to identify differentially expressed genes at selected time points during the recovery process. Bioinformatic analysis revealed two phases of processes: early after stroke (4–7 d post-injury), the spinal transcriptome is characterized by inflammatory processes, including phagocytic processes as well as complement cascade activation. Microglia are specifically activated in the denervated corticospinal projection fields in this early phase. In a later phase (28–42 d post-injury), biological processes include tissue repair pathways with upregulated genes related to neurite outgrowth. Thus, the stroke-denervated spinal gray matter, in particular its intermediate laminae, represents a growth-promoting environment for sprouting corticospinal fibers originating from the contralesional motor cortex. This dataset provides a solid starting point for future studies addressing key elements of the post-stroke recovery process, with the goal to improve neuroregenerative treatment options for stroke patients.

**SIGNIFICANCE STATEMENT** We show that the molecular changes in the spinal cord target tissue of the stroke-affected corticospinal tract are mainly defined by two phases: an early inflammatory phase during which microglia are specifically activated in the target area of reinnervating corticospinal motor neurons; and a late phase during which growth-promoting factors are upregulated which can influence the sprouting response, arborization, and synapse formation. By defining for the first time the endogenous molecular machinery in the stroke-denervated cervical spinal gray matter with a focus on promotors of axon growth through the growth-inhibitory adult CNS, this study will serve as a basis to address novel neuroregenerative treatment options for chronic stroke patients.

## Introduction

Stroke is a leading cause of neurological disability and often impairs motor and sensory systems, resulting in a severely decreased quality of life (Murphy and Corbett, 2009). Functional improvements are most considerable within the first few weeks to months after the incident in human stroke survivors (Cramer, 2008). The chance for functional recovery depends on the stroke size and location, with the integrity of the motor cortex being a determining factor for recovery success (Cramer, 2008). Spontaneous recovery may be observed within the first 3 months after small strokes, but is limited in the case of larger strokes (Cramer, 2008; Ward, 2017). To date, the only treatment option for chronic stroke patients is rehabilitative training, which may result in different degrees of recovery of motor functions (Langhorne et al., 2009). Structural plasticity of spared, intact cortical or corticofugal circuitry has been causally linked to improved outcome in stroke patients (Cramer, 2008) as well as animal models (Murphy and Corbett, 2009; Wahl and Schwab, 2014). After substantial tissue loss, the contralesional corticospinal tract (CST) participates in functional recovery by spontaneous compensatory rewiring in mice, e.g., in the stroke-denervated hemicord on cervical levels (Murphy and Corbett, 2009; Ueno et al., 2012; Bachmann et al., 2014).

This post-stroke compensatory sprouting of intact neurons requires a well organized sequence of events, during which fully integrated and differentiated corticospinal motor neurons (CSMNs) are triggered to grow and to participate in stroke recovery [0–3 d post-injury (dpi); Carmichael and Chesselet, 2002] by initiation and elongation of axonal arbors through the adult growth-inhibitory spinal tissue (7–14 dpi; Carmichael et al., 2005; Li et al., 2010). A functionally relevant circuit is established by interaction and synapse formation with spinal neurons, likely followed by a phase of circuit refinement (28–42 dpi; Ueno et al., 2012; Bachmann et al., 2014; Wahl et al., 2014). Previous studies have unveiled a growth-promoting neuronal intrinsic transcriptome of cortical neurons which sprout horizontally or spinal projecting neurons after stroke or pyramidotomy, potentially enabling them to contribute to functional recovery (Li et al., 2010; Fink et al., 2017). However, less is known about potential target tissue-derived factors, especially in the growth-inhibitory spinal environment, that might influence the reinnervation by the CST of denervated areas after stroke.

Here, we analyzed the transcriptomic profile of the intermediate laminae of the cervical spinal gray matter (GM) representing the main target area of sprouting contralateral CSMNs at selected time points matching the key events of stroke-induced structural plasticity (Carmichael et al., 2005, 2017). We found 955 genes to be differentially regulated in the spinal GM over all time points, although the most pronounced differential regulation was observed at 28 dpi. Results show an early inflammatory phase within the first week after stroke, as well as a more dynamic phase at later stages of recovery, during which upregulation of growth-promoting genes might influence growth and target interaction by the sprouting contralateral CSMNs. The dataset obtained in this study may serve as a starting point for future studies concerning key elements of stroke recovery on the spinal level, including inflammation, synaptogenesis, and circuit reformation.

## Materials and Methods

### 

#### 

##### Experimental design.

The primary aim of this study was to identify molecular factors that influence functional recovery after stroke. In adult mice, the reinnervation of the stroke-denervated cervical hemicord by the contralesional, intact cortex and CST was assessed by anterograde tracing and subsequent quantification. Sham-operated animals were compared with animals that recovered for 1–6 weeks after injury. Animals were randomly distributed into the groups using the R package “blockrand” using two blocks (morning/evening and female/male) to ensure a balance between groups throughout the surgery days and groups. Experimenters were blinded where possible (except for behavioral analyses because of obvious stroke-induced motor deficits). For the anatomical studies, all animals with a stroke ablating the right motor cortex were included in the study. No statistical outliers were excluded. For the transcriptomic study, the target area of the reinnervating contralesional CSMNs was dissected at chosen time points to identify stroke-induced changes in gene expression. Eight animals underwent surgery per time point. Samples were processed simultaneously for RNA extraction. The samples were scored on several quality control criteria, i.e., sufficient ablation of the right motor cortex, deficit in motor behavior, correct location, and size of dissected spinal cord tissue and sufficient RNA quality and quantity. Based on these scores, five samples per time point were chosen for subsequent RNA sequencing. One sample was excluded from the 4 dpi group for further analysis of significantly differentially expressed genes (SDEGs) because it was identified as an outlier in the statistical testing of differential expression.

##### Animals.

All experimental procedures were approved by the veterinary office of the canton of Zurich, Switzerland. A total of *n* = 94 adult C57BL/6J mice (2–3 months, 20–28 g, mixed sex, Charles River Laboratories) were used in this study. For the anatomical study, a total of *n* = 54 animals (22 females, 32 males) were used. For the transcriptomic study, only female mice (*n* = 40) were used to decrease lesion size variability. Animals were housed in groups of four to five under a constant 12 h light/dark cycle with food and water *ad libitum*.

##### Photothrombotic stroke.

For all surgeries, mice were initially anesthetized using 3–4% isoflurane, transferred to a stereotactic frame (Kopf Instruments) and kept at 1–2% isoflurane throughout the surgery. Body temperature was maintained at 37°C on a heating pad. All animals received a unilateral photothrombotic stroke to lesion the right side sensorimotor cortex as previously described (Watson et al., 1985; Bachmann et al., 2014). Briefly, the skull was exposed by a midline incision of the scalp. An opaque template with a defined opening (3 × 5 mm) was aligned to the midline over the right motor and premotor cortex (i.e., −2 to +3 mm A/P, 0–3 mm M/L related to bregma; Tennant et al., 2011). Five minutes after intraperitoneal injection of 0.1 ml Rose Bengal (10 mg/ml in 0.9% NaCl; Sigma-Aldrich), the skull was illuminated with a cold light source (Olympus, KL1500LDC, 150W, 3000K) placed firmly on top of the skull for 10.5 (females, 20–25 g) or 11 min (males, 25–28 g). Control animals received a sham operation without illumination of the skull. Postoperative care included recovery on a heating mat, sustained analgesia provided via drinking water (Novalgin, 2 mg/ml with 5% sucrose) and antibiotic treatment where necessary for 3 d.

##### Anterograde tracing of the forelimb cortex.

The contralesional CST was labeled using the anterograde tracer Biotinylated dextran amines (BDA) (MW 10,000, 10% solution in H_2_O; Invitrogen). Ten days before euthanasia, mice were fixed in a stereotactic frame and the scalp exposed. A craniotomy was performed over the left motor cortex with the dura being kept intact. Five injection sites were chosen at specific forelimb related coordinates (±1.25|−0.5 mm, ±2|−0.5 mm, ±1.25|+0.5 mm, ±2|+0.5 mm, ±1.25|+2 mm, in relation to bregma; Tennant et al., 2011). Two 40 nl injections were applied with a 33 gauge, 10 μl syringe driven by an electrical pump with a flow rate of 6 nl/s at 1 mm and 0.8 mm below the cortical surface. To avoid backflow of the tracer, the needle was kept in position for 2 min after each injection. After the last injection, the animal was sutured and removed from the stereotactic frame.

##### Behavioral testing.

Behavioral tests were performed before surgery (baseline) as well as 4, 7, 14, 28, and 42 d after photothrombotic stroke (dpi) of the right motor cortex. Post-stroke impairment and recovery of forelimb function was assessed using the cylinder test. Forelimb paw touches to the cylinder wall during spontaneous rearing behavior were recorded for 20 min or 30 rears in total (*n* = 10). Paw dragging of the affected limb was scored as previously described (Roome and Vanderluit, 2015) and expressed as the percentage of paw drags divided by total number of paw touches. The horizontal ladder walk test was used as an additional, more sensitive assessment of skilled limb placement (Metz and Whishaw, 2002). Three trials on a 40 cm long ladder with irregularly spaced rungs of 1–2 cm distance were recorded on each testing day (*n* = 48). Skilled forepaw placement on the rungs was scored as previously described (Maier et al., 2008). Blinding was not possible because of obvious stroke-induced motor deficits. However, we followed strict criteria to analyze the motor output: briefly, a full success (1 point) was counted when all four digits were placed correctly in front of the rung. Misplacement of one or more digits or of the palm of the paw was counted as partial success (0.5 points each), whereas a complete misplacement and subsequent slip was counted as no success (0 points). The success score was expressed as percentage of success (sum of success points) in relation to maximal possible outcome (total number of steps taken). The number of foot errors was measured as the number of misplacements divided by the total number of steps taken. All recorded steps were analyzed and no videos were excluded for the analyses to avoid bias toward stroked groups (*n* = 3 videos per animal per test day).

##### Perfusion fixation and tissue processing.

All mice were terminally anesthetized with 3–5% isoflurane followed by injection of pentobarbital (300 mg/kg body weight, i.p.; Streuli Pharma AG). For the anatomical studies, animals were transcardially perfused with Ringer's solution [containing 10^5^ IU/L heparin, (Roche) and 0.25% NaNO_2_] followed by 4% phosphate-buffered formaline containing 5% sucrose. The brain and spinal cord were dissected, postfixed in the same solution for 16 h and transferred to 30% sucrose in PBS for cryoprotection. For the transcriptomic study, animals were transcardially perfused with ice-cold Ringer's solution only. To preserve RNA quality, all following procedures were done rapidly to minimize time between animal kill and tissue collection. Brains and spinal cords were dissected and the intermediate layer of spinal levels C5/C6 was dissected by crude trimming with blades und a dissection microscope. The spinal samples were snap frozen in liquid nitrogen and stored at −80°C until further processing. Adjacent spinal tissue was analyzed for spinal anatomy to ensure correctness of the spinal level. The brains were immersed in 4% PFA overnight before being transferred to 30% sucrose in phosphate buffer (PB) for cryoprotection. Samples were blinded from the point of tissue harvesting.

##### Analysis of lesion completeness.

For the accurate analysis of lesion size, brain cross-sections (40 μm) were stained with cresyl violet and coverslipped with Eukitt. Brain sections at four defined landmarks (1.98, 0.98, −0.22, and −1.34 mm in relation to bregma) were analyzed for depth of the cortical lesion. Transverse spinal cord sections were cut and immunostained with an antibody against the γ-subunit of protein kinase C (PKC) (rabbit anti-PKC-γ, Santa Cruz Biotechnology; 1:200), an established marker for CST fibers (Mori et al., 1990), followed by goat anti-rabbit AlexaFluor 488 (Invitrogen; 1:200). Completeness of the CST lesion on the spinal level was analyzed as percentage of signal in the stroked CST area of the dorsal funiculus in relation to the healthy area of the same section.

##### Quantification of axonal sprouting.

To assess the distribution of BDA-labeled CST fibers within the denervated spinal hemicord, coronal spinal sections of 40 μm thickness were stained on-slide using the nickel-enhanced DAB (3,3′-diaminobenzidine) protocol (VectaStain ABC Elite Kit, Vector Laboratories). Samples of all groups were mixed on each slide to avoid bias induced by the staining procedure. After digitization (Zeiss, Axio Scan.Z1, ×100, opened aperture; three sections/spinal level/animal) obtained sections were blinded again and were fitted into a predefined template of the GM (Watson et al., 2009) using the moving least-squares deformation in FIJI (ImageJ; Schaefer et al., 2006) with five defined landmarks. This alignment allowed for correction of potential tissue shrinkage and stretching because of tissue processing. An intermediate step of manual drawing of the fibers using Photoshop CS6 (Adobe) by an experienced observer blinded to the animal and group number was performed to decrease noise levels of the staining background. A defined grid (20 × 20 panels) was aligned to the GM–white matter boundaries of the ventral horn on one side and the midline on the other. The fiber density was analyzed as gray-scale densitometry along each vertical and horizontal grid line using FIJI (ImageJ). Further analysis with a custom-made automated algorithm in MATLAB (R2015b) determined extended minima of the gray values, where each intersection of a fiber with a grid line resulted in a local minimum and was counted as a fiber. Fiber counts were normalized to an animal-specific normalization factor calculated as the mean gray value of the labeled dorsal CST area of all analyzed sections of the animal divided by the mean gray value of the CST area of all animals in the experiment. False color coded heat-maps for all groups were created with each panel representing the fiber counts at the medial and ventral grid point according to stereologic methods (Schmitz and Hof, 2005). Using this analysis, midline crossing CST fibers were automatically counted approximately each 100 μm and summed up to ∼200 μm bins. Difference maps were created as stroked minus sham maps. Counts of fibers were summed according to laminae predefined in the spinal cord atlas (Watson et al., 2009) to obtain a laminar specific analysis. To count fibers at the midline and the CST–GM border, sections of spinal levels C5/C6 were digitalized (Zeiss, Axio Scan.Z1, ×100; three sections/level/animal, *z*-stack of 10 images/slice with 1 μm distance) and fibers were counted manually using FIJI.

##### RNA purification and RNA-sequencing.

Total RNA was prepared from all samples simultaneously by using the NucleoSpin RNA XS Kit (Macherey-Nagel) under the protocols of the manufacturer with an additional TRIZol/chloroform extraction step because of the high lipid content of the spinal cord. The quality of the isolated RNA was determined with a Qubit 1.0 Fluorometer (Life Technologies) and a Bioanalyzer 2100 (Agilent). Only samples with a 260 nm/280 nm ratio between 1.8 and 2.1 and a 28 S/18 S ratio within 1.5–2 were further processed. The TruSeq RNA Sample Prep Kit v2 (Illumina) was used in the succeeding steps. Briefly, total RNA samples (100 ng) were ribo-depleted using Ribo Zero Gold (Epicenter) and then fragmented. The fragmented samples were reverse transcribed to cDNA, end-repaired and polyadenylated before ligation of TruSeq adapters containing the index for multiplexing. Fragments containing TruSeq adapters on both ends were selectively enriched by PCR. The quality and quantity of the enriched libraries were validated using Qubit 1.0 Fluorometer and the Caliper GX LabChip GX (Caliper Life Sciences). The product is a smear with an average fragment size of ∼260 bp. The libraries were normalized to 10 nm in Tris-Cl 10 mm, pH 8.5 with 0.1% Tween 20. The TruSeq SR Cluster Kit v4-cBot-HS (Illumina) was used for cluster generation using 10 pm of pooled normalized libraries on the cBOT. Sequencing was performed on the Illumina HiSeq 4000 (single end, 125 bp) using the TruSeq SBS Kit v4-HS.

##### RNA-seq data analysis.

All statistical analysis was performed using the R environment for statistical computing (R v3.4.1) with Bioconductor (Huber et al., 2015) and dedicated packages. Alignment to the mouse reference genome (mm10) was performed by SALMON (v0.8.1), followed by gene quantification with Tximport (v1.0.3) using standard settings (Soneson et al., 2015). Expression level estimation was reported as counts per million (CPM) together with confidence intervals for each sample, although genes with an expression level of ≤5 were excluded to avoid technical artifacts (*n* = 28967 genes excluded). As a result, 16,995 transcripts were included in the analysis. Raw gene counts were normalized using edgeR (Robinson et al., 2010) and differential expression was expressed as the average CPM of one group divided by the average CPM of the sham operated animals. SDEGs were defined by a false discovery rate (FDR) < 0.05 and a log fold-change > 1.5 (*n* = 955 SDEGs). Hierarchical clustering was conducted in R (package “pheatmap”, Euclidean distance). To study the biological functions of relevant clusters identified by hierarchical clustering, we applied gene ontology (GO) enrichment analysis on respective gene sets using the Cytoscape (http://www.cytoscape.org/) plug-in BiNGO v2.44 (Maere et al., 2005) with standard settings and a FDR set to 0.05. Enriched GO terms were visualized by red nodes, where color represents the corresponding FDR-adjusted enrichment *p* value (*q* value) and size represents the gene set size of the according GO term. To further validate the obtained dataset, genes of the morphogenesis related pathways (e.g., anatomical structure development, morphogenesis of a branching structure) were cross-referenced to the literature to focus on genes that were previously connected to neurite outgrowth.

##### Microglia activation.

Free-floating sections of spinal tissue were quenched, permeabilized with 1% Triton in blocking buffer (10% normal goat serum and 1% bovine serum albumin in 0.05 m tris-buffered saline) and subsequently incubated with primary antibody (rabbit anti-Iba1, 1:1000; Wako) in blocking buffer overnight. The sections were subsequently incubated in secondary antibody (biotinylated goat anti-rabbit, 1:200) in blocking buffer. Sections were stained using the DAB protocol (VectaStain ABC Elite Kit, Vector Laboratories). Sections were thoroughly washed, mounted on slides and air dried overnight. Before coverslipping, sections were dehydrated using increasing ethanol concentrations and xylol incubation. Microglial activation was analyzed using morphological features. Slides were digitalized (Zeiss, Axio Scan.Z1, ×100; three sections/spinal level/animal, *z*-stack of 5 images/slice with 2 μm distance) and Iba1^+^ cells were marked in the GM by an experienced observer blinded to the group. A FIJI-based algorithm was used to select 20 Iba1^+^ cells within the GM in a semirandom manner that allowed selection of cells randomly but in an even distribution throughout the GM. This was achieved by placing a grid of a random spacing (50–100 μm) and selecting the closest marked cell to each intersection point. If this resulted in <20 cells, the spacing between the grid lines was decreased and new cells were selected. For each selected cell, the soma diameter and the longest process was measured to calculate the activation index as longest process of the cell divided by the diameter of the soma; thus, smaller values represent a more activated state of the cell. Three regions were defined as regions-of-interest: ventral and dorsal horn, the CST within the dorsal funiculus as well as the former CST projection area, which was defined as the area of dense projection of the healthy CST. For this, anterograde tracing was performed on healthy animals (3 sections/5 animals) and BDA^+^ fibers were identified in the spinal GM. The CST projection area was defined to outline the area of 80% of maximum staining intensity. The obtained data were further processed in MATLAB (R2015b) to classify the cells according to their spatial distribution and to obtain false color-coded heat-maps of Iba1^+^ cell activation and population density.

##### Neurite outgrowth assay.

Candidate proteins selected from the screen were tested for their neurite growth promoting capacity on neurons growing in presence of growth inhibitory adult spinal cord protein extract. To obtain spinal cord protein extracts, rats were killed by decapitation. The spinal cord was dissected and immediately homogenized using ice-cold extraction buffer (3.7% CHAPS and 5 mm EDTA in PBS) containing 1× HALT TM protease and phosphatase inhibitor (ThermoFisher Scientific). Homogenized tissue was incubated on ice for 30 min and centrifuged four times for 15 min at 13,000 × *g* at 4°C. Supernatant was aliquoted and snap-frozen in liquid nitrogen. The protein concentration was determined using a Bradford assay. N1E-115 mouse neuroblastoma cells were obtained from ATCC and maintained at 37°C in a humidified atmosphere with 5% CO_2_ in DMEM supplemented with 10% fetal bovine serum, 2% l-glutamine and 1% penicillin-streptomycin. Neuron-like differentiation was induced by switching the medium to serum-free Neurobasal supplemented with 2% l-glutamine and 1% PenStrep. Cells were plated in differentiation medium at a density of 10,000 cells/cm^2^. After 24 h, the cells were treated with spinal cord extract and candidate factors and left to grow for another 24 h. The following proteins were used at the indicated concentrations: 1 ng/ml transforming growth factor β1 (TGF-β1; R&D Systems, catalog #7666-MB-005/CF), 100 mm semaphorin 6A (Sema6a; R&D Systems, catalog #9017-S6-050), 100 mm Netrin G2 (Ntng2; R&D Systems, catalog #2744-NG-050), and 100 mm growth differentiation factor 7 (GDF7; R&D Systems, catalog #2744-NG-050). The assays were stopped by the addition of 4% PFA at room temperature for 15 min. The cells were then counterstained with Coomassie solution (0.25% Coomassie brilliant blue R250, 50% MeOH, 10% HoAC) for 5 min, followed by two consecutive washes with PBS and stored at 4°C. Randomized images were acquired on a ScanR HCS microscope (Olympus) equipped with an UPLSAPO 10× objective. Mean neurite outgrowth was quantified in ImageJ by applying a grid to the pictures and counting intersections of neurites with the grid lines and total cell bodies and calculating the ratio thereof (Rønn et al., 2000). Each experiment was conducted in three technical replicates.

##### *In situ* hybridization.

Freshly dissected, unfixed spinal tissue was collected as described above and directly snap-frozen on dry ice. Sections (16 μm) were cut on a cryostat at −20°C and stored at −80°C. *In situ* gene expression was assessed using either the RNAScope protocol (Advanced Cell Diagnostics, RNAscope 2.5 HD Assay-RED) or viewRNA (ThermoFisher Scientific, QVT 1-Plex Assay Kit) according to the manufacturer's protocol. Briefly, in both kits, slices were fixed in 4% PFA for 20 min before being hybridized to the probe (RNAScope: TGF-β1, catalog #407751; viewRNA: Sema6a, catalog #VB6-3116963-VCP; Ntng2, catalog #VB1-3046339-VT), which were further amplified using branched DNA amplification methods. The probes were detected using a fast red detection kit. Sections were counterstained with DAPI and digitalized (Zeiss, Axio Scan.Z1, ×200). For analysis, *n* = 3 sections per animal per group were randomly selected based on their tissue intactness. Quantification of mRNA expression was done by densitometric analysis as the percentage of white signal over black background in four defined regions (CST in the dorsal funiculus, ventral horn, dorsal horn, and intermediate layers) of the denervated hemicord after applying gene-specific thresholds, which were defined for five randomly selected images of the dataset and averaged.

##### Statistical analysis.

Statistical analysis was performed with Prism 7.0 (GraphPad Software) and R v3.4.1. For statistical tests within groups over time, ordinary one-way ANOVA (one-way ANOVA) or linear mixed models (LMMs) followed by Dunnett's multiple-comparisons (MC) test were used. To detect differences between groups and within groups over time and for comparison of more than two groups over time, two-way ANOVA with repeated measures followed by Tukey multiple-comparisons test was used. The threshold for significance for all experiments was set at *p < 0.05. Smaller *p* values were represented as **p < 0.01 and ***p < 0.001. Other symbols might be used to indicate two comparisons in one graph. In bar graphs, all data are plotted as mean ± SEM. In box plot graphs, data are represented as median ± 25th percentile (box) and min/max (whiskers). In the neurite outgrowth assay, data were normalized to PBS controls. In all graphs, dots represent individual animals.

##### Accession codes.

The accession number for the raw sequencing data reported in this paper is NCBI GEO: GSE128623.

## Results

### Motor behavior partially recovers after unilateral motor cortex stroke in young adult mice

**Figure 1.**
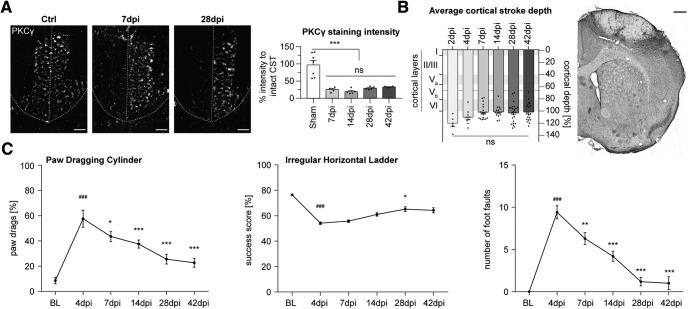
Large unilateral cortical strokes lead to motor function impairments that recover over time. ***A***, Immunoreactivity for the CST marker PKC-γ in the cervical spinal cord. Representative pictures show the intact CST on both sides of a sham-operated animal (left) and an absence of signal on the left (lesioned) side in a stroked animal at 7 dpi and 28 dpi (middle and right). Scale bar, 50 μm. Quantification of signal strength for CST in relation to the intact CST of the healthy hemicord (*n* = 5/group). ***B***, Quantification of cortical stroke depth for all animals in all experimental groups. Location of CSMNs in layer V is indicated by a dotted line. The sensorimotor area was specifically injured (Nissl staining at 7 dpi, representative picture). Scale bar, 500 μm. ***C***, Forelimb motor impairment assessed by paw dragging cylinder task (*n* = 10) and horizontal ladder task (*n* = 50). #Indicates comparison to baseline levels, *Indicates comparison to 4 dpi.

We induced a large microthrombotic stroke in the right sensorimotor cortex of 2- to 3-month-old mice of both sexes by intraperitoneal Rose Bengal injection and skull illumination. Strokes were similar in dimensions in all animals (Fig. 1*A*,*B*) and did not differ between sexes (data not shown). PKC-y staining on spinal levels showed a successful >80% ablation of the corresponding corticospinal tract with no significant difference between experimental groups (*p* > 0.8082, one-way ANOVA with MC; Fig. 1*A*). The lesion reached into deep cortical layers across all animals used in the subsequent studies with no significant differences between experimental groups (*p* = 0.4717, one-way ANOVA; Fig. 1*B*).

To assess functional performance, we measured motor impairment and subsequent recovery using the paw dragging cylinder test and the horizontal ladder task. In the paw dragging cylinder test, paw drags were counted during spontaneous rearing behavior. Shortly after injury, the number of paw drags increased significantly (baseline–4 dpi: ###*p* < 0.001, LMM with MC), although a gradual spontaneous recovery was observed over the following 6 weeks (4–7 dpi: *p = 0.03399, 4–42d pi: ***p < 0.001, LMM with MC; Fig. 1*C*). Using a more sensitive test, the horizontal ladder task, we addressed fine motor skills of the forelimbs by scoring forelimb rung placements. At 4 dpi, all stroked animals showed a strong deficit in limb placement and targeting as detected by a reduction in success score and an increase in the number of forelimb foot faults compared with baseline (baseline–4 dpi: ###*p* < 0.001, LMM with MC; Fig. 1*C*). The motor performance gradually improved over the course of the following 6 weeks (success score, 4–28dpi: *p = 0.00995; foot faults, 2–42 dpi: ***p < 0.001, LMM with MC). Both behavioral tests show that ablation of the right sensorimotor cortex resulted in an impairment of skilled motor function, although a partial spontaneous recovery can be observed in the course of 6 weeks after the injury.

### The contralesional corticospinal tract sprouts and reinnervates the stroke-denervated hemicord

**Figure 2.**
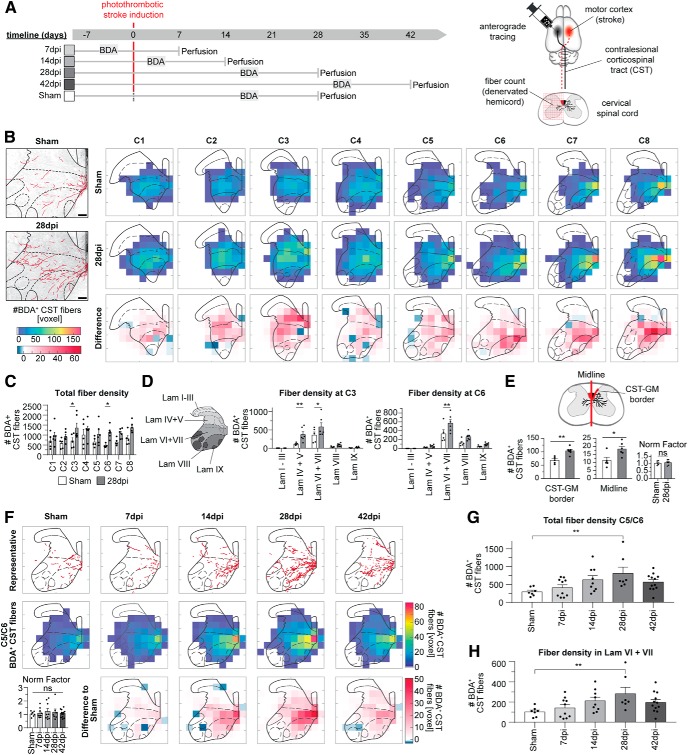
Contralesional corticospinal fibers cross the spinal cord midline on all cervical levels after large unilateral stroke. ***A***, Experimental timeline of the study. Adult C57BL/6 mice (*n* = 54) received a large unilateral cortical stroke. Anterograde tracing of the CST was performed 10 d prior perfusion and sprouting was analyzed at 7, 14, 28, and 42 dpi. Control animals received a sham operation. BDA^+^ fibers were counted in the cervical spinal cord. ***B***, Representative sections of the cervical spinal cord with highlighted BDA^+^ fibers of the contralesional CST 28 d after stroke compared with sham animal. Scale bar, 100 μm. Average false color coded heat maps of BDA-labeled fiber counts on cervical levels C1–C8 of sham animals (top row; *n* = 5) and stroked animals at 28 dpi (middle row; *n* = 6). Difference map (bottom row) represents the difference between stroke to sham groups (average of stroked group minus average of sham). Increased fiber density is depicted in red, decreased fiber density in blue colors. ***C***, Quantification of total fiber density across the whole GM across cervical segments. ***D***, Laminar analysis (based on the schematic representation of the Rexed laminae) on spinal levels C3 and C6. ***E***, Stereological intersection counting of BDA^+^ fibers at the CST–GM border and the spinal cord midline. ***F***, Average false color-coded heat maps of BDA^+^ fiber counts on cervical levels C5/C6 after 7, 14, 28, and 42 dpi, and after sham operation (*n* = 7–13/group). Top row, Representative drawings of fiber innervation. Middle row, Average false color-coded heat map of BDA-labeled fiber counts across groups. Bottom row, Difference map (average of stroked group minus average of sham). Increased fiber density is depicted in red, decreased fiber density in blue colors. ***G***, ***H***, Quantification of total fiber counts of the whole GM (***G***) and within lamina VI+VII (***H***) of cervical segments C5/C6. *P < 0.05, **P < 0.01, ***P < 0.001.

In mice, a significant degree of spontaneous reinnervation of the stroke-denervated cervical hemicord by sprouting of contralesional corticospinal fibers can be detected 28 d after lesion (Ueno et al., 2012; Bachmann et al., 2014). To assess the temporal and spatial distribution of this contralesional corticospinal input, we injected the anterograde tracer BDA into the intact, contralesional forelimb motor cortex of stroked and sham-operated animals (Fig. 2*A*). First, we semiautomatically quantified BDA^+^ fibers in the stroke-denervated hemicord 28 dpi across all cervical levels of the spinal cord (Fig. 2*B*). The “side switch” of descending corticospinal projections was present on all levels of the cervical spinal cord, but fiber growth was markedly increased after stroke in cervical segments C3 and C6 as seen by total fiber counts (C3: *p = 0.0475, C6: *p = 0.0394, two-way ANOVA with MC; Fig. 2*C*). Using a detailed laminar analysis, we show that newly formed projections targeted primarily laminae IV–V and VI+VII in C3 (Lam IV+V: **p = 0.0082, Lam VI+VII: *p = 0.0275, two-way ANOVA with MC) and C6, respectively (Lam IV+V: **p = 0.0010, two-way ANOVA with MC; Fig. 2*D*).

Increased innervation by the contralesional CST might be established by increasing terminal arbors of the pre-existing crossed or uncrossed projections that can be found in mice (Fig. 2*B*; representative picture of Sham-operated animal) or by formation of new projections from the intact side that grow across the spinal segmental midline (Weidner et al., 2001; Lindau et al., 2014). To investigate the origin of the stroke-induced fiber increase, we counted BDA^+^ fibers exiting the CST on spinal levels C5/C6 within the intact hemicord at the border between the GM and the CST in the dorsal funiculus, as well as at the spinal cord midline (dorsal and ventral commissure; Fig. 2*E*). An increased number of BDA^+^ fibers was found to branch off the CST into the GM (**p = 0.0018, unpaired *t* test) with a concomitant increase in fiber counts at the spinal cord midline (*p = 0.0233, unpaired *t* test). These results suggest an increase in new projections arising from the CST of the intact hemicord with extensive branching in the appropriate layers of the denervated hemicord.

To address the temporal profile of reinnervation by contralesional CSMNs, we quantified BDA^+^ fibers at cervical levels C5/C6 in the stroke-denervated hemicord at 7, 14, 28, and 42 dpi (Fig. 2*E–G*). We observed a gradual increase in fiber counts up to 28 dpi when compared with sham-operated animals (Sham, 28 dpi: **p = 0.005, one-way ANOVA with MC), with a subsequent reduction at 42 dpi (Sham, 42 dpi: *p* = 0.2015; Fig. 2*E*,*F*). This temporal projection pattern was also seen in the intermediate laminae VI+VII (Sham, 28 dpi: **p = 0.0092, one-way ANOVA with MC; Fig. 2*G*).

### The stroke-denervated intermediate laminae of the cervical spinal cord show differential gene expression

**Figure 3.**
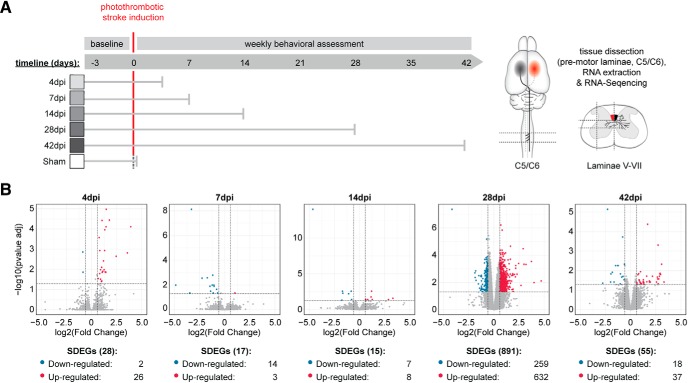
Comparison of gene expression between stroke-denervated and sham spinal cords at selected time points after injury reveals injury related changes in lamina IV-VII on spinal levels C5/C6. ***A***, Experimental timeline of the study. Animals received a large unilateral cortical stroke. Tissue samples were collected by microdissection of the intermediate laminae on spinal level C5/C6 at 4, 7, 14, 28, and 42 dpi (*n* = 8/group). Control animals received a sham operation. ***B***, Volcano plots display the log_2_ fold-change versus -log_10_ of the adjusted *p* value for each differentially expressed gene after comparison of the respective time point to the sham group (*n* = 5/group). The cutoff criteria of 1.5 log fold-change and FDR of < 0.05 are indicated by dotted lines in each panel. Downregulated SDEGs are depicted in blue, upregulated SDEGs in red. The full list of genes as well as their expression patterns at the different time points can be found in the GEO repository (accession code GSE128623).

After confirming the intermediate laminae VI+VII on spinal level C6 as the target area densely reinnervated by contralesional CSMNs, we sought to identify the transcriptomic profile of this spinal tissue at selected time points reflecting key time points of stroke recovery (4, 7, 14, 28, 42 dpi). For this, we micro-dissected the intermediate laminae of the stroke-denervated hemicord on spinal level C5/C6 for RNA extraction (Fig. 3*A*). Samples were further processed on the Illumina 4000 HiSeq platform. Alignment of reads to the mouse transcriptome showed a successful mapping to the genome (∼70%, data not shown) comparable to previous studies on spinal tissue (Shi et al., 2017). Using a false discovery rate (FDR) of < 0.05 and a minimum *log*_2_-fold change of 1.5, a total of 955 SDEGs were identified, although the most pronounced differential regulation (891 SDEGs) was seen at 28 dpi (Fig. 3*B*).

**Figure 4.**
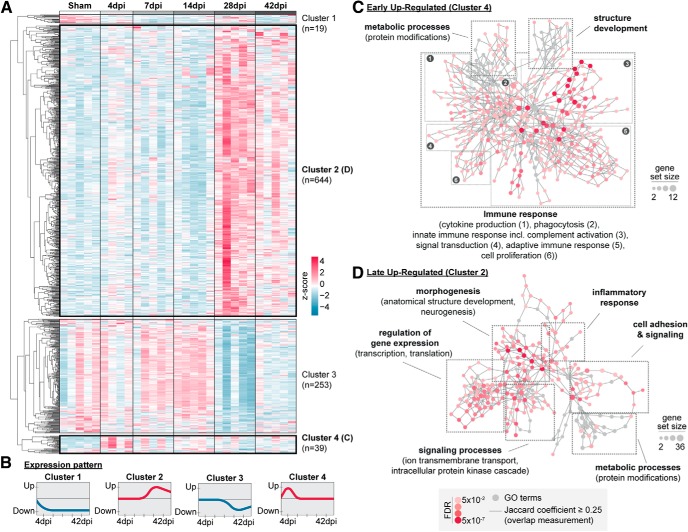
Hierarchical clustering of significantly differentially expressed genes reveals two phases of biological function during the time course of recovery after stroke. ***A***, Scaled expression values of all 955 differentially expressed genes are shown for each group (Sham, 4, 7, 14, 28, and 42 d after stroke, FDR < 0.05) with red values indicating an upregulation of the gene, whereas blue values indicate a downregulation. Columns represent single samples/animals, rows represent single genes. Ontologic hierarchical clustering among rows identified four clusters (correlation distance measure). The list of genes of the different clusters can be found in Figure 4-1. ***B***, Patterns of gene expression after stroke identified by clustering: stroke-induced long-term downregulated genes (Cluster 1), late upregulated genes (Cluster 2), late downregulated genes (Cluster 3), and early upregulated genes (Cluster 4). ***C***, ***D***, Network visualization of the GO enrichment analysis using BiNGO for the 39 early upregulated genes of Cluster 4 (***C***) and for the 644 late upregulated genes of Cluster 2. Refer to Data Figure 4-2, for a tabular overview of the GO term analysis and the SDEGs involved (***D***). Red nodes depict enriched GO terms, where color represents the corresponding FDR-adjusted enrichment *p* value and size reflects the gene set size of differentially regulated genes. Refer to Figure 4-3, for a tabular overview of the GO term analysis and the SDEGs involved.

Unbiased hierarchical clustering of all SDEGs identified four clusters representing different expression patterns (Fig. 4*A*,*B*, and Fig. 4-1). Cluster 1 (19 SDEGs) covered genes that were downregulated upon stroke induction (4 dpi) and stayed at lower levels persistently. Cluster 2 comprised 644 SDEGs that were upregulated at late time points (28 and 42 dpi), whereas genes of Cluster 3 (253 SDEGs) were downregulated at late time points. Last, early upregulated genes (39 SDEGs) clustered together in Cluster 4. We performed additional GO term analysis for clusters 2 and 4 to identify factors that might elicit sprouting or guide the reinnervating fibers coming from the contralesional CSMNs. Network visualization of the SDEGs of Cluster 2 (Fig. 4*C*, and Fig. 4-2) revealed several hubs to be differentially regulated, including regulation of gene expression and cell adhesion and signaling. Notably, SDEGs of Cluster 2 were found enriched in morphogenic biological processes such as anatomical structure development (Sema6A, Notch3, Syngap1, TGF-βR3, Itga3, LIF, GDF7, PlxnA1) and morphogenesis of a branching structure (TGF-β1, Sox9, Pax2, GDF7, PlxnA1). In Cluster 4 (Fig. 4*D*, and Fig. 4-3), the major hub was associated with inflammatory processes such as cytokine production (CD74, LAG3, FCGR3, CLEC7A, FCGR2B), phagocytosis (FCGR3, CLEC7A, FCGR2B), and complement activation (C1qa- C1qc, C4b, CSF1R, FCGR2, TREM2). Interestingly, selected markers of the recently identified novel subtype of microglia (“disease associated microglia”; Keren-Shaul et al., 2017) were found among these early upregulated genes (CSF1R, GRN, TREM2, MPEG, CLEC7A).

10.1523/JNEUROSCI.2571-18.2019.f4-1 Figure 4-1 Hierarchical clustering of the SDEGs expressed in the spinal target area. Download Figure 4-1, XLSX file

10.1523/JNEUROSCI.2571-18.2019.f4-2 Figure 4-2 Go Term Analysis of Cluster 2 (late upregulated SDEGs). Download Figure 4-2, XLSX file

10.1523/JNEUROSCI.2571-18.2019.f4-3 Figure 4-3 Go Term Analysis Cluster 4 (early upregulated SDEGs). Download Figure 4-3, XLSX file

### The immune response in the stroke-denervated spinal GM early after stroke

**Figure 5.**
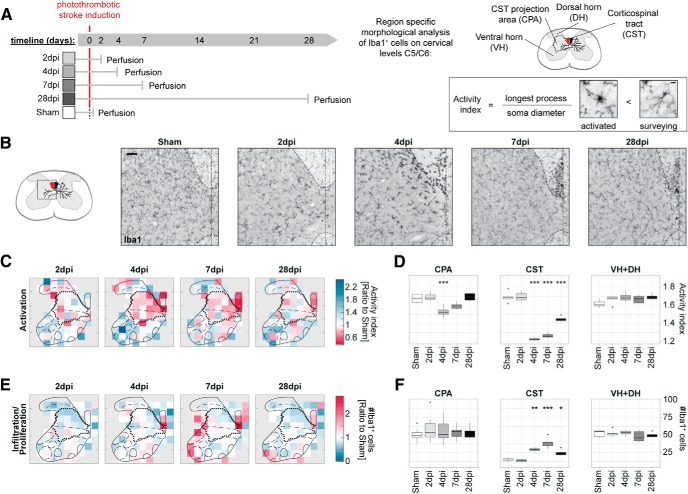
Microglia/macrophage (Iba1^+^ cells) activation and infiltration/proliferation on spinal level C5/C6 after stroke. ***A***, Experimental timeline of the study. Animals received a photothrombotic stroke and were killed 2, 4, 7, or 28 dpi thereafter. Control animals received a sham operation (*n* = 5/group). Morphological analysis was conducted on Iba1^+^ stained transverse sections. Activation index was calculated as the longest process of the cell divided by the soma diameter. Lower values indicate an activated morphology, higher values a morphology toward non-activated, resident tissue surveying states. Scale bar, 10 μm. ***B***, Representative pictures of Iba1 immunostaining of CST and CPA in spinal cord GM (boxed area) in Sham and stroked animals at 2, 4, 7, and 28 dpi. Scale bar, 50 μm. ***C***, Difference map of the activation index of Iba1^+^ cells at selected time points (fold-change to Sham maps). Red values depict increased activation, while blue values indicate the ramified surveying state based on morphology. ***D***, Quantification of activation index in different regions across the experimental timeline. ***E***, Difference map of number of Iba1^+^ cells at selected time points (fold-change to Sham group). Increase in cell number is depicted in red; reduction in blue colors. ***F***, Quantification of Iba1^+^ cells corresponding to region across the experimental timeline. *P < 0.05, **P < 0.01, ***P < 0.001.

To further investigate the two phases defined by the hierarchical clustering, we first addressed the response of CNS-associated immune cells within the stroke-denervated spinal GM at early time points. The morphology of Iba1^+^ microglia and macrophages was evaluated at 2, 4, 7, and 28 dpi (Fig. 5*A*) in selected areas within the spinal cord, e.g., the ventral horn (VH) and dorsal horn (DH), the CST area within the dorsal funiculus (CST) as well as the projection area of the stroke-damaged CST [CST projection area (CPA)]. We defined an activation index based on the feature of microglia to retract their processes concomitant with an increase in soma diameter upon activation (Davis et al., 1994; Stence et al., 2001; Fig. 5*A*). We found an increased number of Iba1^+^ cells with an activated morphology in the CST area in the dorsal funiculus at 4 dpi that persisted up to 28 dpi (***p < 0.0001, one-way ANOVA with MC; Fig. 5*B–D*). In the CPA, Iba1^+^ cells also portrayed an alerted phenotype at 4 dpi, however, this activation was only observed transiently and was regulated back to baseline levels within 28 d (***p < 0.0001, one-way ANOVA with MC; Fig. 5*D*). Infiltration of blood born monocytes or division of resident microglia was assessed by the number of Iba1^+^ cells in the regions of interest and revealed an increase of Iba1^+^ cells in the CST after 4 dpi (**p = 0.001, one-way ANOVA with MC; Fig. 5*B*,*E*,*F*), whereas levels remained increased until 28 dpi (*p = 0.0263, one-way ANOVA with MC). Infiltration/proliferation, however, was not observed in the CPA (Sham, 4 dpi: *p* = 0.421, one-way ANOVA with MC) or in the ventral and dorsal horn of the spinal GM at any of the measured time points, suggesting that the activated cell type in the CPA is predominantly microglia.

### The spinal GM expresses tissue-derived growth-promoting factors at 4 weeks after stroke

**Figure 6.**
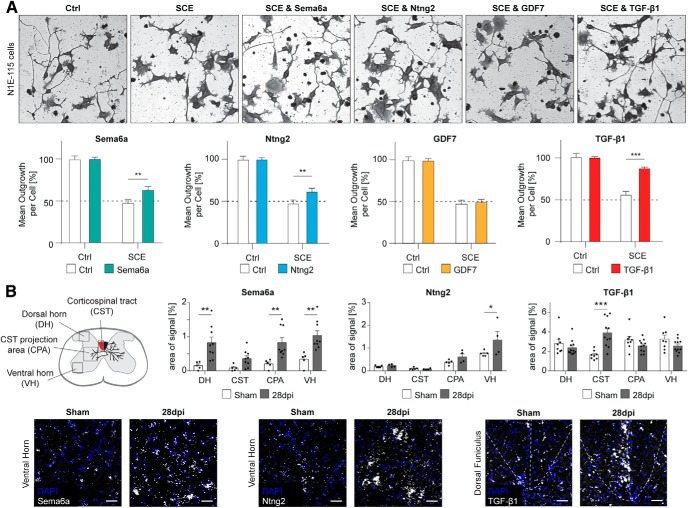
The late upregulated genes Sema6a, Ntng2, and TGF-β1, but not GDF7, partially rescue neurite outgrowth under inhibitory conditions and are expressed in the spinal GM at late stages after stroke. ***A***, Representative pictures of N1E-115 cells 24 h after addition of control medium (Ctrl), SCE, or the combination of SCE and Sema6a/Ntng2/GDF7/TGF-β1 protein, respectively. Quantification of mean neurite outgrowth per cell is normalized to medium control condition. ***B***, *In situ* hybridization with subsequent analysis of signal intensity of the mRNA in four selected regions within the stroke-denervated hemicord 28 dpi and after sham operation. Representative pictures of *in situ* hybridization for Sema6a and Ntng2 in the ventral horn and TGF-β1 in the dorsal funiculus. Scale bar, 50 μm. *P < 0.05, **P < 0.01, ***P < 0.001.

Based on the GO term analysis, four candidates of the late upregulated genes of Cluster 2 (Fig. 4*C*) were chosen for further analysis of their growth-regulating potential in an *in vitro* neurite outgrowth assay: Sema6a, Ntng2, GDF7, and TGF-β1. To assess the potential neuronal growth promoting role of these candidates in an inhibitory environment, we used a well defined brain derived neuronal cell line (N1E-115). After induction of differentiation, N1E-115 cells extend neurite-like processes. Addition of spinal cord extract (SCE) inhibits this extension in a dose-dependent manner, simulating the growth inhibitory effect of the adult spinal cord. Sema6a, the mRNA of which we found to be upregulated 1.5-fold 4 weeks after stroke, was recently found to have a growth-promoting effect on PC12 cells (Tian et al., 2017) and is important for the correct wiring of the CST during development (Faulkner et al., 2008; Rünker et al., 2008). In our assay, Sema6a did not have a direct neurite inducing effect under control conditions. However, combined treatment with SCE resulted in a partial rescue of neurite outgrowth from SCE inhibition (**p = 0.0031, two-way ANOVA with MC; Fig. 6*A*). This growth-promoting effect under inhibitory conditions was also seen for Ntng2, which we found upregulated 1.8-fold in the spinal target area of sprouting CSMNs in our transcriptomic screen (**p = 0.0044, two-way ANOVA with MC). Ntng2, a vertebrate-specific member of the UNC-6/netrin family, promotes neurite outgrowth of primary cortical neurons (Nakashiba et al., 2002) and plays a role in sensorimotor behavior such as optokinetic reflex or the hanging wire test (Zhang et al., 2016). The bone morphogenic protein (BMP)/TGF-β superfamily has recently been implicated in post-stroke structural plasticity (Li et al., 2015). We found two members of this family to be upregulated in the stroke-denervated spinal GM: GDF7 and TGF-β1. GDF7 did not affect neurite outgrowth in either control or SCE conditions. On the other hand, TGF-β1, the mRNA of which we found 1.5-fold upregulated, showed a strong growth enhancing effect antagonizing the growth-inhibitory SCE (***p < 0.0001, two-way ANOVA with MC). These data are in line with previous studies where TGF-β1 administration increased re-elongation of injured hippocampal neurons as well as primary cortical neurons (Abe et al., 1996; Li et al., 2015). Thus, we show the presence of effective neurite growth-inducers or -modulators that are upregulated 4 weeks after stroke in the stroke-denervated hemicord.

We further addressed the localization of expression of the three candidates that influenced neurite outgrowth within the spinal tissue using *in situ* hybridization in cervical level 5/6 at 28 dpi. The amount of mRNA of Sema6a, assessed as the percentage of signal over background, showed an ubiquitous increase throughout the spinal GM of the stroke-denervated hemicord compared with sham-operated control animals (DH: **p = 0.004, VH: **p = 0.0028, CPA: **p = 0.0081, two-way ANOVA with MC; Fig. 6*B*). Thus, Sema6a might positively regulate the growth of CSMN sprouts into the stroke-denervated spinal target area. Ntng2 showed an increased expression only in the ventral horn in *in situ* hybridization (VH: *p = 0.0101, two-way ANOVA with MC). Based on the enrichment of Ntng2 mRNA in large cell bodies within the ventral horn, Ntng2 might primarily be expressed by motoneurons in the ventral horn at 28 dpi. This may suggest a role for Ntng2 as an attractive guidance cue for sprouting CST fibers, steering their path into the ventral, motor quadrant of the spinal GM. Interestingly, an upregulation of TGF-β1 mRNA was primarily seen in the CST area of the dorsal funiculus (***p < 0.0001, two-way ANOVA with MC). This localization might indicate the secreted factor TGF-β1 as a prime candidate for growth initiation and axon elongation/arborization in the stroke-denervated spinal cord.

## Discussion

We used a unilateral large stroke to investigate changes in the transcriptome of the spinal cord from 4 d to 6 weeks after injury, a time window during which the stroke affected CST degenerates and fibers of the contralesional CST initiate sprouting, grow across the spinal midline, and reinnervate the denervated hemicord, mainly laminae IV–VII in the mouse. Transcriptomic profiling of this target area revealed stroke-induced differential expression of a total of 955 genes, with the most pronounced changes seen at Day 28 after stroke. Hierarchical clustering and GO term analysis showed an enrichment of inflammation-related pathways in the early days after stroke, whereas tissue-regeneration related pathways were highlighted at later stages. For three upregulated genes of the late phase, Sema6a, Ntng2, and TGF-β1, the potential to overcome spinal cord extract mediated growth-inhibition was shown in an *in vitro* neurite outgrowth assay, suggesting their role as growth-promoters in the spinal GM after stroke.

The formation of new circuits within the brain is one of the fundamental elements of post-stroke recovery that can be observed across several species, including rodents, primates, and humans (Buetefisch, 2015; Murata et al., 2015; Morecraft et al., 2016; Carmichael et al., 2017; Ward, 2017). Which areas participate in this post-stroke rewiring may primarily depend on size and location of the ischemic insult (Dijkhuizen et al., 2003; Hsu and Jones, 2006; Zhu et al., 2010). After considerable tissue loss, the contralesional cortex is recruited by “side-switching” part of its projections. In rats, contralesional, crossed CSMNs have been shown to form bilateral projections within the first 2 weeks after stroke, followed by retraction of the original, contralateral arbor (Lindau et al., 2014). In mice, contralesional CSMNs increase the arborization of their pre-existing midline-crossing or uncrossed ipsilaterally projecting segmental axons or extend new, midline-crossing fibers (Ueno et al., 2012; Wahl et al., 2014). Here, we observed a twofold increase in CST branches entering the GM from the intact-side CST in the dorsal funiculus and an equally large increase in the number of fibers crossing through the midline commissures into the denervated hemicord. These observations suggest a segmental switch of these CSMNs, perhaps similar to the initial innervation of the CST during development (Canty and Murphy, 2008). This rewiring of the contralesional CST in rodents was shown to be a crucial mediator of motor recovery, as cortical ablation or selective chemogenetic silencing of reinnervating CSMNs led to a reappearance of the initial stroke-induced behavioral deficits (Bachmann et al., 2014; Wahl et al., 2014).

The molecular mechanisms by which this fiber growth is induced and guided in the spinal target area have not been studied so far. Previous gene profiling *in vivo* studies addressing post-stroke recovery focused on the cortex at early time points in a quest to find candidates to boost neuroprotection (Lu et al., 2003; Rickhag et al., 2006; Sarabi et al., 2008; Hori et al., 2012; Zeng et al., 2017) or were restricted to the stroke penumbra, where an interpretation of the data in regards to growth-promoting factors may be difficult because of additional stroke-induced processes such as inflammation, scar formation, or angiogenesis (Keyvani et al., 2002; Carmichael et al., 2005; Krüger et al., 2006; Buga et al., 2014). In our study, we addressed whole-tissue gene expression changes within the stroke-denervated hemicord GM, far remote from the acute processes around the cortical lesion site. One expectation was that the transcriptomic profile of the spinal target area should reflect soluble, membrane-bound and extracellular matrix factors attracting and guiding sprouting fibers of the CST but also of other supraspinal command centers such as the brainstem (Bachmann et al., 2014). At 28 d after stroke, we found several previously indicated growth-modulators to be upregulated in the stroke-denervated hemicord. Itga3 as a proposed receptor for Netrin-1 (Nikolopoulos and Giancotti, 2005) might facilitate and enhance cell-contact-dependent axon guidance within the stroke-denervated hemicord. LIF, a cytokine modulating glial and neuronal function during development, has been implicated in axonal regeneration after spinal cord and optic nerve injury (Blesch et al., 1999; Leibinger et al., 2009). Administration of LIF *in vitro* leads to increased neurite outgrowth and promotes elongation but not arborization (Cafferty et al., 2001). *In vivo*, LIF administration leads to increased levels of neurotrophins such as NT-3 (Blesch et al., 1999), potentially caused by activation of glial cells as well as peripheral inflammatory cells after injury (Holmberg and Patterson, 2006; Ostasov et al., 2015). Furthermore, four upregulated candidates were addressed in this study as potential stroke-induced growth-promoters: Sema6a, Ntng2, GDF7, and TGF-β1. The latter strongly antagonized the neurite outgrowth inhibition of spinal cord extract *in vitro*, whereas Sema6a and Ntng2 had weaker growth promoting effects. Additional differentially upregulated genes of this screen may provide further candidates as modulators of structural plasticity. Interestingly, we also found an upregulation of growth-inhibitors such as PlxnA1, Srgap1, or Sox9 (Nawabi et al., 2010; Delloye-Bourgeois et al., 2015; McKillop et al., 2016) concomitant with a downregulation of well established growth-promoters such as BDNF or metallothioneins I and II (Barde et al., 1978; Chung et al., 2013). BDNF has repeatedly been implicated as a growth-promoter in *in vitro* assays (Gillespie et al., 2001; Salie and Steeves, 2005; Santos et al., 2016) and in models of stroke (Ueno et al., 2012); BDNF effects can further be promoted by rehabilitation training (Ploughman et al., 2009). However, the role of BDNF on sprouting of the corticospinal tract remains controversial (Lu et al., 2001). In the present study of spontaneous recovery we found a late downregulation of BDNF mRNA levels in the stroke-denervated hemicord, corroborating previous studies of protein levels in the cervical spinal cord (Sist et al., 2014). These findings might reflect a switch from pro-growth mechanisms toward stabilization or pruning of new synaptic connections at 28–42 dpi (Ueno et al., 2012). Although the current study focuses on genes involved in growth initiation, axon elongation, and guidance, our dataset will also reflect genes involved in synaptogenesis and synaptic plasticity at the later phases of functional recovery.

A recent study by Fink et al. (2017) showed that 4 weeks after unilateral transection of the CST, Nogo-A deficient sprouting contralateral CSMNs upregulated genes related to pathways of axonal growth facilitation. Thus, the CSMNs intrinsically employ a growth-promoting transcriptome at 4 weeks after injury in line with the findings of a late growth-promoting tissue environment found in the present study. These late changes of transcriptional regulation might also reflect arborization processes of the reinnervating sprouts within the target area. Further extracellular cues for reinnervating sprouts might be present outside of the reinnervating area, e.g., within the healthy hemicord, where they could steer the reinnervating fibers over the midline into the denervated hemicord at earlier time points. In the future, novel candidates should also be tested in an *in vivo* model of CNS injury such as stroke or spinal cord injury for their potential to induce compensatory or regenerative sprouting of motor-related pathways such as the corticospinal tract. Given the intricate post-stroke re-wiring which requires both spatial and temporal control (Wahl and Schwab, 2014), such interventions need to be carefully timed to validate candidates as novel growth enhancing therapeutics. TGF-β1, which was highlighted in this study as a growth-modulator, also promotes tissue fibrosis and might negatively impact liver and immune function when applied systematically (Akhurst and Hata, 2012). Although neutralizing antibodies often provide a useful tool, viral manipulations or inducible transgenic knock-outs of the respective candidate may be preferable to study the specific role of a novel candidate on motor recovery.

Both at early as well as later stages after stroke, immune-related pathways were differentially regulated. Additionally, we showed an activation of Iba-1^+^ cells in the stroke-denervated hemicord explicitly at 4 dpi. Iba1 is an often used marker that stains both microglia as well as infiltrating macrophages unspecifically (Greter et al., 2015). However, based on the morphological changes and cell numbers observed in the intermediate spinal laminae, we suggest that the subpopulation of activated Iba1^+^ cells primarily comprises microglia. This may, in future studies, be highlighted using novel and more specific microglia staining marker (Bennett et al., 2016) or microglia-specific transgenic reporter mice (Jung et al., 2000; Buttgereit et al., 2016).

An early inflammatory response within the first week after stroke suggests a role for microglia/macrophages in phagocytosis of cellular and myelin debris related to the degradation of the CST and its terminals (Thomalla et al., 2005; Weishaupt et al., 2010). Indeed, microglial triggering receptor expressed on myeloid cells-2 (TREM2), a factor we found upregulated at early time points, has been shown to facilitate the clearance of debris in the absence of inflammation (Takahashi et al., 2005). Additionally, TREM2 recently was identified as a positive regulator for M2 microglia polarization while simultaneously negatively regulating M1 polarization (Zhang et al., 2018), priming the microglia toward a phenotype aiding in tissue repair (Sierra et al., 2013; Ma et al., 2017). It has been proposed that inflammatory cells, especially microglia, may be linked to regenerative success because of the production of growth-promoting factors and anti-inflammatory cytokines, which may stimulate sprouting of axons and myelin repair (Neumann et al., 2009). In future experiments, it will be of high interest to investigate the activated microglia in this injury model for their cell-specific transcriptome (Hirbec et al., 2017), especially given the recently identified subtype of disease associated microglia (Keren-Shaul et al., 2017). Our model and dataset may provide a tool to study the link between debris clearance and regeneration of axons in future studies.

The results of the present study have further defined the role of the spinal cord in motor recovery after stroke. The early inflammatory response in the stroke-denervated hemicord, most likely induced by the degenerating CST synapses and fibers is suggested to contribute to the initiation of structural plasticity and sprouting by some of the genes and pathways described here. At 4 weeks after injury the stroke-denervated hemicord, in particular its intermediate laminae, expresses growth factors and represents a growth-promoting environment for, e.g., sprouting CST fibers originating from the contralesional motor cortex. Here, increased structural and synaptic plasticity of contralesional CSMNs might directly enhance post-stroke motor recovery.

Detailed analyses of the dataset in regard to novel growth-promoters expressed at late stages will provide further insights into potential candidates and pathways that influence the post-stroke recovery processes and may lead to novel neuroregenerative treatment options for stroke patients.
